# Prescription opioid use and 12-month depression trajectories

**DOI:** 10.1016/j.psychres.2026.117096

**Published:** 2026-03-18

**Authors:** Jeffrey F. Scherrer, Joanne Salas, Aniketh Naidu, Scott Secrest, Lauren Wilson, Celeste Pappas, Patrick J. Lustman, Brian Ahmedani, Ryan W. Carpenter, Lisa R. Miller-Matero, Mark D. Sullivan

**Affiliations:** a Department of Family and Community Medicine, Saint Louis University School of Medicine, 1008 S. Spring SLUCare Academic Pavilion, 3rd Floor, St. Louis, MO 63110, USA; b Advanced HEAlth Data (AHEAD) Research Institute, Saint Louis University School of Medicine, 3545 Lafayette Ave, 4th Floor, St. Louis, MO 63104, USA; c Department of Health and Clinical Outcomes Research, Saint Louis University School of Medicine, 3545 Lafayette Ave, 4th Floor, St. Louis, MO 63104, USA; d Center for Health Policy and Health Services Research and Behavioral Health Services, Henry Ford Health, One Ford Place, Detroit, MI 48202, USA; e Department of Psychiatry, Washington University School of Medicine, 4320 Forest Park Blvd, Suite 301, St. Louis, MO 63108, USA; f Department of Psychology, University of Notre Dame, 390 Corbett Family Hall, Notre Dame, IN 46556, USA; g Department of Psychiatry and Behavioral Science, University of Washington School of Medicine, 1959 NE Pacific Street, Seattle, WA 98195, USA; h Department of Psychiatry and Behavioral Neuroscience, Saint Louis University School of Medicine, 1438 South Grand Blvd. St. Louis, MO 63104, USA

**Keywords:** Pain, Opioid, Epidemiology, Depression, Trajectory, Prospective, Cohort

## Abstract

**Introduction:**

Retrospective cohort studies indicate prescription opioids increase risk for depression. No prospective cohort studies have modeled prescription opioid use and depression trajectories. We determined whether daily vs. non-daily prescription opioid use was associated with more severe and worsening depression.

**Methods:**

Participants with a new period of prescription opioid use were recruited from two health care systems. Those who enrolled were invited to complete twelve-monthly surveys over a year follow-up. Depression was measured with the PHQ-9, and opioid use was self-reported. From the 760 participants with 3 or more monthly surveys, we computed PHQ-9 latent growth trajectories. A 3-class solution best fit the data and revealed a severe-increasing depression trajectory, a moderate-stable, and a low-decreasing trajectory.

**Results:**

Participants were 53.6 ± 11.7 years of age, 68.5% were female and 70.5% identified as white race. Daily vs. non-daily opioid use was not significantly associated with depression trajectories. Higher emotional support (OR=0.92:95%CI:0.89–0.94) and ability to participate in social roles (OR=0.96; 95%CI:0.93–0.99) protected against, while pain related functional impairment (OR=1.39; 95%CI: 1.11–1.74), more pain sites (OR=1.20; 95% CI: 1.10–1.31) and smoking (OR=2.38; 95%CI:1.27–4.47) increased the likelihood of severe-increasing depression compared to low-decreasing depression.

**Conclusions:**

The lack of an independent link between daily opioid use and worsening or more severe depression in this sample suggests that increased rates of depression in daily opioid-exposed patients may be a result of high risk patients receiving daily opioids rather than a direct effect of the opioid exposure. Screening for factors related to depression should improve safer opioid prescribing.

## 1. Introduction

Results from retrospective cohort studies using medical record and medical claims data have consistently shown that long-term opioid therapy (LTOT) is associated with up to a 2-fold greater risk for new depression diagnoses (Scherrer et al., 2016; Scherrer et al., 2014; Wilson et al., 2019). A more recent retrospective cohort study revealed that the risk for new onset depression among those on LTOT is limited to patients with daily or near daily opioid use (Scherrer et al., 2022). Evidence from a Mendelian randomization study revealed a causal relationship between prescription opioid use and depression (Rosoff et al., 2021). Of note, these studies have shown this relationship between LTOT and depression is independent of pain and opioid use disorder (OUD).

In contrast to retrospective cohort studies using administrative medical record data, mixed results have come from three prospective cohort studies of prescription opioid use and new onset depression (Scherrer et al., 2015; Smith et al., 2015; Von Korff et al., 2017). One prospective cohort study observed increasing dose was followed by increasing odds of depression in follow-up (Scherrer et al., 2015) while two others have found no significant association between dose or duration of opioid use and new depression episodes (Smith et al., 2015; Von Korff et al., 2017). To our knowledge, only one study has modeled both opioid use frequency and dose. Specifically, Von Korff and colleagues found no association between minimal/no use, intermittent/lower dose, and regular/higher dose opioid use and odds of depression at 4 and 12-month follow-up (Von Korff et al., 2017). The investigators suggested the large number of lower dose, intermittent users in the sample might have precluded detecting a significant association. One limitation of these existing studies is that they were not designed to measure how depression symptoms progress following the start of a new period of prescription opioid use. Because there are months to year long delays between the time a patient first experiences depression symptoms and when they are diagnosed (Benasi et al., 2021), existing retrospective studies may miss subclinical depression and are vulnerable to misclassification of undiagnosed depression following LTOT. In addition, existing work has evaluated prescription opioids and new onset depression, and not depression trajectories over time, regardless of baseline depression status.

Modeling trajectories of depression may reveal when and how quickly depression severity can change following LTOT. This is important in management of chronic non-cancer pain given the well-established bi-directional relationship between pain and depression. If we observe that opioid exposure accounts for this relationship, then this study will inform safer selection of patients for opioid therapy. If daily opioid use is associated with patterns of depression, then results could identify the time since opioid initiation at which depression symptoms tend to worsen or become clinically meaningful. If more frequent opioid use is associated with worsening depression, then providers and prescribing guidelines should include repeated screening for mood disorder throughout opioid therapy.

The present study uses repeated depression assessment in a cohort starting a new period of prescription opioid use to determine if opioid use frequency is associated with trajectories of depression symptoms. We hypothesize that more frequent opioid use will be associated with more severe depression that is either stable or worsening.

## 2. Methods

### 2.1. Setting

The prospective Prescription Opioids and Depression Pathways cohort study, henceforth termed the “Pathways” study, began recruitment in 11/2019, and ended enrollment 11/2022. Patients starting a new period of prescription opioid use were recruited to the Pathways study from two healthcare systems, Saint Louis University’s academic medical practice, St. Louis, Missouri and Henry Ford Health, Detroit, Michigan. Both systems provide comprehensive inpatient and outpatient care. All study procedures were reviewed and approved by the Saint Louis University and Henry Ford Health IRBs.

### 2.2. Study design

The Pathways study protocol and participant characteristics at baseline have been previously reported (Scherrer et al., 2020; Secrest et al., 2024). In brief, Pathways is a prospective cohort study with survey administration at baseline, 6- and 12-month follow-up, and an additional twelve, brief, monthly surveys. The overarching purpose of the Pathways study was to evaluate biopsychosocial outcomes related to prescription opioid use.

### 2.3. Eligibility

Patients were eligible to enroll in the Pathways cohort study if they were 18 to 80 years of age, had an electronic health record (EHR) indicating no current cancer related pain (confirmed via survey), and had started a new period (no opioid use in the 3 months before baseline) of prescription opioid use. We required patients to have prescriptions totaling 30 to 90 days of opioid use at baseline to enrich the sample with participants likely to remain on prescription opioids during follow-up. Prescriptions could be from any provider type and included all opioid medications prescribed for pain. The reason for which opioids were prescribed was for any non-cancer condition and may have changed during follow-up.

### 2.4. Participants

As part of obtaining informed consent, patients were asked to release their EHR data for the 12 months prior to baseline and for the 12 months of follow-up. Those who did not consent to EHR release were exited from the interview. Of the 1047 enrolled, 81.4% completed the 6-month follow-up survey and 74.8% the 12-month follow-up.

Participants who completed the baseline survey were invited to complete up to 12 brief, monthly surveys taking an average of 5-min to complete. Monthly assessments were the PEG-3, Patient Health Questionnaire - 9 (PHQ-9) and an update on self-reported prescription opioid use. The PEG measures pain intensity [P], interference with enjoyment of life [E] and interference with general activity [G] (Krebs et al., 2009) and the PHQ-9 is a well-validated depression measure (Kroenke et al., 2001b). Participants reported opioid dose and dosing frequency. The present study uses data from 760 participants who completed the baseline survey, at least 3 monthly assessments to allow for trajectory modeling, and complete data on pertinent baseline variables included in this analysis.

Participants were given a $50 gift card for each baseline, 6-month and 12-month survey and $10 gift cards for each monthly survey completed. Those who completed all assessments were eligible for a $1000 raffle at 6- and 12-month follow-up.

### 2.5. Outcome

Depression trajectories were computed using monthly PHQ-9 measures (Kroenke and Spitzer, 2002; Kroenke et al., 2001a). Scores below 5 indicate minimal depression, between 5 and 9 is mild depression, 10 to 14 is moderate, 15 to 19 moderate/severe and 20 to 27 is severe depression (Kroenke and Spitzer, 2002; Kroenke et al., 2001b). A minimum of 3 monthly surveys were required to compute trajectories.

### 2.6. Exposures

Frequency of opioid use was categorized into daily vs. not-daily. We asked patients how often they take their opioid (days per week) and based on patient report of their dosing regimen, we categorized those who were daily users vs. anything less than daily. Both daily and non-daily users could have a second opioid prescription to take as needed for breakthrough pain. In this case, we still used number of days per week to define daily vs. non-daily use. Baseline opioid type and dose were converted to morphine milligram equivalent (MME) dose by using self-report unit dose and number taken per day. We began by following the Middle-Aged/Seniors Chronic Opioid Therapy (MASCOT) study (Von Korff et al., 2017) to create 4 exposure groups: 1) non-daily use and <50 MME; 2) non-daily use and ≥50 MME; 3) daily use and <50 MME and 4) daily use and ≥50 MME. After computing the distribution of this variable by trajectory membership, it was clear that there were too few participants in the non-daily use and ≥50 MME trajectory to use the 4-level variable as the exposure. The most common pattern of opioid use was daily and <50 MME (47.2%), followed by non-daily and <50 MME (26.5%), daily and ≥50 MME (13.8%) and non-daily and ≥50 MME occurred in 1.8% of the sample. Thus, sample size was too small to use this 4 group exposure and instead we modeled daily vs. non-daily opioid exposure use. We leverage evidence that only daily or near daily opioid use among patients on LTOT is associated with new onset depression (Scherrer et al., 2022), to determine if daily, compared to non-daily, prescription opioid use is linked to depression trajectories. Thus, the primary exposure is daily vs. not-daily opioid use at baseline. We did not restrict the sample to those with or without lifetime depression as that could generate an unrepresentative sample of chronic non-cancer pain patients with LTOT.

### 2.7. Baseline covariates

Covariates were measured at baseline and included demographics, social support, pain measures, non-opioid pain therapies, psychiatric, substance use disorders and problem opioid use. Sociodemographic variables included age, biological sex, and race (White vs. non-white).

The PROMIS SF v2.0-Emotional Support 4a scale measured emotional support with higher scores indicating more support (“PROMIS SF v.2.0-Emotional Support 4a scale. http://www.healthmeasures.net/search-view-measures?task=Search.search. Accessed 2020 Apr 22,”) The PROMIS Ability to Participate in Social Roles and Activities v.2 measured perceived barriers, not pain specific, to leisure, family, work and activity with friends (Hahn et al., 2010). Higher scores indicate greater ability to perform social roles and activities. Using published reference populations in PROMIS scoring manuals, scores on each scale were standardized into a T-score, with mean of 50 and standard deviation of 10.

The number of pain sites, pain severity and pain-related interference with activities were assessed with the Brief Pain Inventory (Cleeland, 1991; Keller et al., 2004) ICD-10 diagnostic codes measured arthritis, back/neck pain, musculoskeletal pain, neuropathies, migraine/headache, fibromyalgia, and chronic pain obtained from electronic health records in the year prior to baseline. ICD-10 codes for these conditions are shown in the Appendix, e-Table 1.

Non-opioid treatments included gabapentin, NSAIDs, muscle relaxers, and steroids. Non-pharmacological treatments were physical therapy and interventional pain management were measured with prescription orders and Current Procedural Terminology (CPT) codes in the EHR in the year prior to baseline completion (see Appendix e-table 1 for CPT codes). EHR data included prescriptions for antidepressant medications and benzodiazepines.

We also adjusted for participant reported smoking, psychiatric and substance use disorders assessed at baseline. Lifetime smokers were those who reported ever smoking 100 or more cigarettes in their lifetime. Based on current smoking status (“do you now smoke every day, some days, or not at all?”), participants were classified as never/past vs. current smokers (e.g., smoke some days or every day). The Primary Care Posttraumatic Stress Disorder (PC-PTSD) screener was administered to obtain probable PTSD diagnosis (score of at least three) (Prins et al., 2016), and the GAD-7 measured generalized anxiety disorder, with a score of at least 10 (at least moderate) indicating anxiety (Spitzer et al., 2006). We adjusted for Vital Exhaustion (VE) using the Maastricht Vital Exhaustion brief form. VE is “unusual fatigue, increased irritability and feelings of demoralization” with higher scores indicate worse VE with a score of ≥10 indicating high vital exhaustion (Meesters and Appels, 1996). The Prescribed Opioids Difficulty Scale (PODS) measured psychosocial problems that patients attributed to opioid use and patient concerns about opioid use. Higher scores indicate greater problems with opioids, including side effects and perceived misuse symptoms. Scores ≥16 were considered a positive PODS score (Banta-Green et al., 2010).

### 2.8. Eligibility for current analyses

From the 1047 baseline participants, 842 had 3 or more monthly assessments, with 835 having at least three valid PHQ-9 scores. After removing those with missing baseline variables, there were 760 participants in the analytic sample (i.e., 91% complete case analysis among those with at least three PHQ-9 scores). We tested if response bias was present by comparing participant characteristics between the entire baseline cohort and those with three or more monthly surveys. As shown in Appendix e-table 2, the only difference was for current smoking, which was significantly, albeit modestly, more prevalent (*p* = 0.028) in the entire sample (26.8%) vs. those with 3 or more monthly surveys (23.4%).

### 2.9. Analytic approach

The first analytic phase created distinct PHQ-9 score trajectories across the 12-months of follow-up using MPlus Version 8.8 to conduct growth mixture modeling (GMM) (Jung and Wickrama, 2008; Muthen and Muthen, 2000; Muthén and Shedden, 1999, 1998–2011). GMM is a general, more flexible extension of latent class growth analysis, allowing intercept and slope parameters to vary across individuals within a class, with heterogeneity captured with random effects. The best fitting model was determined by diagnostics such as AIC, sample size adjusted BIC, entropy, interpretability of classes (i.e., > 5% prevalence), and the Lo-Mendell-Rubin (LMR) adjusted likelihood ratio test comparing k vs. k-1 classes (Jung and Wickrama, 2008; Nylund et al., 2007). Final classes were described using estimated slope and intercept parameters. GMM uses a full information maximum likelihood (FIML) approach to missing data (i.e., missing monthly PHQ-9 scores). FIML uses all available data, even from partial cases, to estimate model parameters and maximizes the likelihood function for each individual case. Missing data is treated as a feature, not error, and FIML provides unbiased parameter estimates under the assumption of missing at random (MAR).

After class enumeration, SAS v9.4 (SAS Institute, Cary, NC) was used for the remaining analyses. Bivariate analyses comparing distributions of baseline variables by depression trajectories and by daily opioid use used chi-square tests, *t*-tests, ANOVA, and negative binomial regression (for pain site count). Tukey’s post-hoc comparisons were conducted when applicable. Multinomial logistic regression models, using the categorical pain trajectory class as the outcome and daily opioid use as the main exposure, were computed. Covariates associated with daily opioid use and depression trajectories at *p* < 0.10 were included in adjusted models. Odds ratios and 95% confidence intervals were calculated.

### 2.10. Results

Cohort characteristics at baseline are shown in Table 1. The sample, on average, was 53.6 ± 11.7 year of age, 68.5% were female and 70.5% were White race. About two-thirds of the sample were daily opioid users at baseline. Also, Appendix e-table 3 shows the distribution of each type of opioid a patient self-reported at baseline. The most prevalent opioids were hydrocodone (48.7%), tramadol (38.3%), and oxycodone (30.8%), and 26.4% of patients endorsed at least two different types of opioids at baseline.

**Fig. 1.**
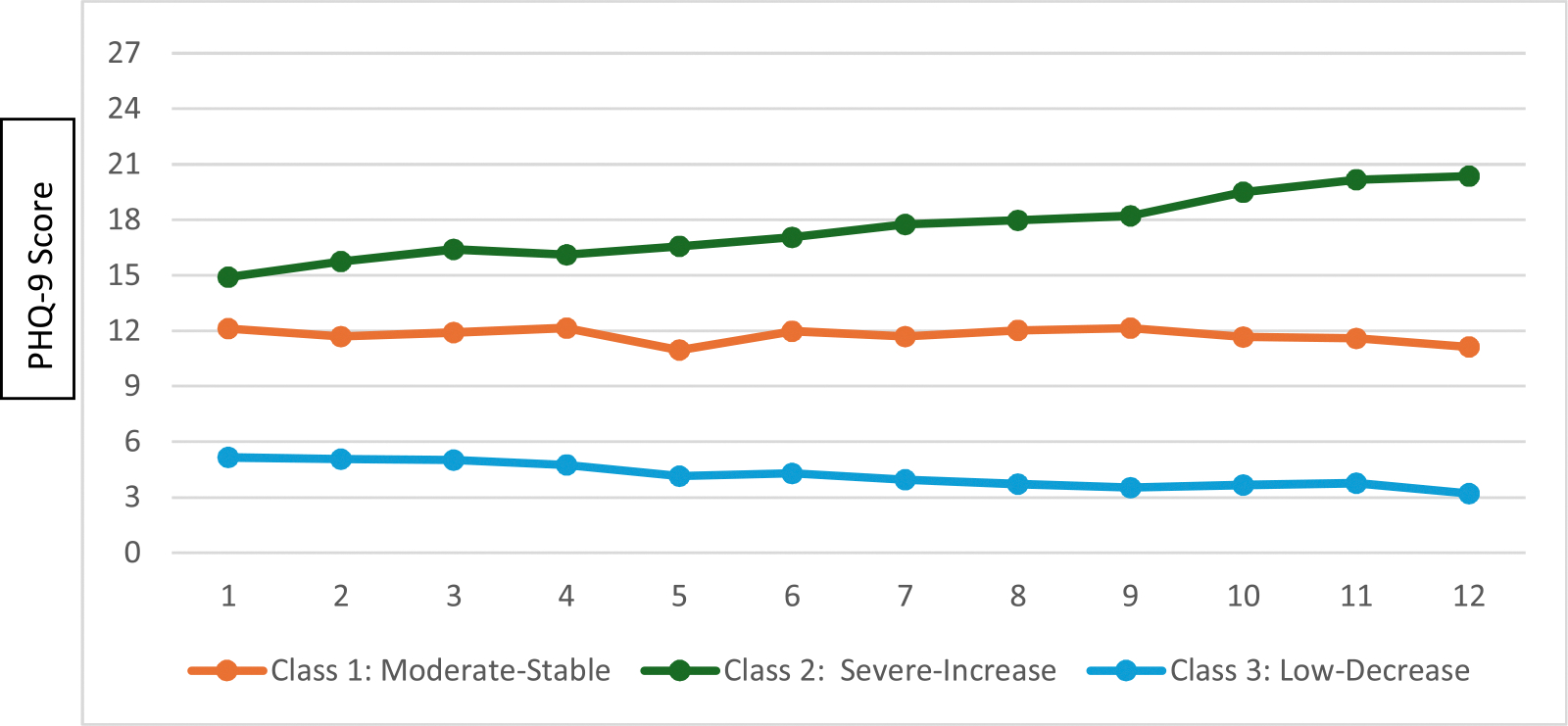
Depression trajectories from three or more monthly PHQ-9 assessments.

GMM goodness-of-fit statistics in Appendix, e-table 4 showed that a 3-class solution best fit the data. The LMR test comparing 4 vs. 3 classes was non-significant indicating no gain in model fit by adding a fourth class. Fig. 1 illustrates the depression trajectories from the best fitting model and Table 2 shows observed Month 1 and Month 12 means as well as GMM estimated slope and intercept parameters. Class 1 (24.9% of the sample) was characterized by “stable-moderate depression” trajectory; Class 2 (9.1%) indicated an “increasing-severe depression” trajectory; and Class 3 (66.0%) was best described as “decreasing-low severity depression.” The moderate stable trajectory had an estimated intercept of 11.5 and a slope of −0.03 (*p* = 0.48). The severe-increasing trajectory estimated intercept was 14.3, with a significant 0.5 point increase in PHQ score per month (slope=0.5, *p* < 0.0001). Finally, the low-decreasing trajectory had an estimated intercept of 5.4, and a significant monthly decrease of 0.2 points per month (slope= −0.2, *p* < 0.0001).

The association between baseline participant characteristics and depression trajectories is presented in Table 3. Age was significantly (*p* = 0.0002) associated with depression trajectories and Tukey’s post-hoc tests revealed significantly older age among the decreasing-low severity trajectory compared to other trajectories. Emotional support (*p* < 0.0001) and ability to participate in social roles were significantly (*p* < 0.0001) related to trajectory membership. Emotional support significantly differed between each trajectory with lowest support among increasing-severe, intermediate level of support in stable-moderate and highest support in the decreasing-low depression trajectory. Ability to participate in social roles was greatest in the decreasing-low depression trajectory as compared to the other two trajectories.

The mean number of pain sites was significantly (*p* < 0.0001) higher in the increasing-severe, followed by the stable-moderate and lowest in the decreasing-low depression trajectory. Average pain severity (*p* < 0.0001) and pain interference (*p* < 0.0001) scores were highest in the increasing-severe, followed by the stable-moderate and lowest in the decreasing-low depression trajectory. Diagnoses for back pain, fibromyalgia, neuropathy, and migraine/headache were each significantly associated with trajectory membership, (p-value range: *p* = 0.018 to 0.0002).

Antidepressant and gabapentin prescriptions were most common among the stable-moderate, followed by increasing-severe and least prevalent among the decreasing-low depression trajectory (*p* < 0.0001), and benzodiazepine prescriptions were most prevalent in the increasing-severe and stable-moderate trajectories and least common in the decreasing-low trajectory (*p* < 0.0001).

Current smoking, probable PTSD, and GAD were significantly (*p* < 0.0001) associated with trajectory membership with the highest prevalence of these variables in the increasing-severe and stable-moderate trajectories and much less common among the decreasing-low depression trajectory. Those with a positive PODS score were most prevalent in the increasing-severe and stable-moderate group and much less common in the decreasing-low trajectory (*p* = 0.002). Last, VE was significantly associated with trajectory membership (*p* < 0.0001). Over 60% of participants in the increasing-severe trajectory were VE positive, while 38.1% of the stable-moderate and 9.2% of the decreasing-low groups had VE.

The distribution of participant characteristics by daily vs. non-daily opioid use is shown in Table 4. Male sex, (*p* = 0.009) and White race (*p* = 0.016) were more prevalent among daily users. Ability to participate in social roles was lower in the daily opioid users (*p* < 0.0002). Having more pain sites (*p* < 0.022), greater pain severity (*p* = 0.0007) and worse pain related interference (*p* < 0.0001) were all positively associated with daily opioid use. Back pain (*p* = 0.003) and musculoskeletal pain (*p* = 0.002) were significantly more prevalent among daily, as compared to non-daily, opioid users. Use of physical therapy (*p* < 0.0001), interventional pain procedures (*p* < 0.0001), gabapentin, and muscle relaxer prescriptions were significantly (*p* < 0.0001) more prevalent in daily compared to non-daily users. Smoking and history of SUD were both positively associated with daily opioid use (*p* < 0.0001). Having a positive PODS score was more common in daily users (*p* < 0.0001).

Results from multivariate, multinominal regression are shown in Table 5, with the decreasing-low depression trajectory as the common referent group. A 1-point increase in emotional support and ability to participate in social roles was associated with a 4–5% decreased odds of a stable-moderate vs. decreasing-low depression trajectory (OR=0.96 and 0.95, respectively). Similarly, each 1-point increase in emotional support or ability to participate in social roles was associated with a 4–8% relative decrease in the odds of increasing-severe vs. decreasing-low depression trajectories (OR=0.92 and 0.96, respectively). Each additional pain site was associated with a 17% increased odds and 20% increased odds in membership in the stable-moderate and increasing-severe trajectories compared to the decreasing-low trajectory, respectively. Smoking was also associated with a 1.76 (95%CI:1.14–2.72) times greater odds in membership in the stable-moderate and 2.38 (95% CI:1.27–4.47) times greater odds in membership in the increasing-severe trajectories compared to the decreasing-low trajectory. Physical therapy use was markedly less likely in the stable-moderate decreasing-low depression trajectories (OR=0.55; 95%CI:0.36–0.84). Prescriptions for gabapentin (OR=1.61; 95%CI:1.07–2.42) and a positive PODS score (OR=1.66; 95%CI:1.02–2.71) were associated with an increased odds for membership in the stable-moderate vs. decreasing-low depression trajectories. Greater pain related interference was the only covariate significantly associated with increasing-severe vs. decreasing-low trajectory membership, with each 1-point unit increase associated with a 39% increased odds (OR=1.39; 95%CI=1.11–1.74) of being in the severe increasing trajectory.

## 3. Discussion

Among patients with non-cancer pain who completed 30 to 90 days of prescription opioid use at baseline, we observed three PHQ-9 depression trajectories over a 12-month follow-up period. These included a stable, moderate depression trajectory; an increasing-severe depression trajectory; and a decreasing-low severity depression trajectory. Daily opioid use was not associated with the trajectories, and contrary to our hypothesis, daily use did not increase risk for membership in the more severe depression trajectories.

The fully adjusted model identified several non-opioid patient characteristics associated with increased risk for stable-moderate depression. These factors included more pain sites, gabapentin prescription, current smoking, and more patient psychosocial problems attributed to opioids and greater concerns about opioid use (i.e., PODS positive). Risk for being in moderate or severe depression trajectories was inversely associated with higher emotional support and ability to participate in social activities. Overall, smoking was the factor most strongly related to moderate and severe depression trajectories. These results suggest that characteristics of patients who receive daily opioids, rather than pharmacological exposure to opioids itself, account for the increased depression risk among daily opioid recipients.

We observed a positive relationship between smoking and moderate and severe depression trajectories. This is consistent with a systematic review which found 73% of studies on tobacco and depression support an association of current smoking and depression (Fluharty et al., 2017). A separate systematic review found evidence that smoking was linked to future depression (Fluharty et al., 2017). Compared to never smokers, current smokers among Veterans Health Administration patients was associated with over a 56% greater odds of having an opioid prescription (Bastian et al., 2017) which is similar to findings in the general population (Encinosa et al., 2025) and compared to never smokers, current smokers used 30% of opioid prescriptions and had 6 times the relative risk of persistent opioid use (Encinosa et al., 2025). Smoking is also a risk factor for chronic pain (LaRowe and Ditre, 2020). Efforts to incorporate smoking cessation in pain management, during opioid taper and treatment of OUD may improve outcomes.

To our knowledge there are no existing studies of prescription opioid use and depression trajectories. The most similar study to the present work is secondary analyses of a clinical trial comparing Naltrexone vs Buprenorphine for Opioid Treatment (XBOT) for persons with OUD (Vest et al., 2023). Over a 2-year treatment trial, three (HAM-D) depression trajectories were identified as high recurring, persistently high and low decreasing depression scores. Relapse was most prevalent in the persistently high trajectory. Results are consistent with the present study in that most patients with OUD had mild depression symptomology (Vest et al., 2023). In a separate cohort study of persons seeking treatment for non-medical OUD, three depression trajectories were identified characterized by moderate-severe stable, moderate-decreasing and mild to no depression symptoms (Ellis et al., 2022). While OUD treatment does not equate to prescription opioid use, the course of depression is generally the same.

Pain related functional interference and ability to participate in social roles were inversely associated with worse depression trajectories. This is consistent with existing research with non-cancer pain as the primary exposure. Compared to no depression, among primary care patients with musculoskeletal pain, pain related interference had a robust positive association with persistent depression and gradual depression recovery, and lack of emotional support was linked to a 5 times greater risk for persistent depression as compared to no depression (Rzewuska et al., 2015). These findings are consistent with the present results. Social support is critical to limiting depression and other psychiatric comorbidities which may in turn reduce prescription opioid use (Sullivan et al., 2024). Group therapy providing social support was the center of one the most successful opioid reduction interventions studied to date (Sandhu et al., 2023).

Depression trajectories from the Health and Retirement study also reveal pain-related functional impairment as a risk factor for chronic depression vs. no or limited depression symptoms (Zhu et al., 2014). Depression trajectories in a cohort followed during the COVID pandemic revealed a severity spectrum with the severe depression group being most likely to been treated with opioids (Angarita-Fonseca et al., 2024).

There are over a dozen retrospective studies (Leung et al., 2022), including Mendelian randomization research (Rosoff et al., 2021) indicating a causal relationship between prescription opioids and new depression episodes. However, this does not mean findings are contradictory because the present study suggests there are clinical factors not available in medical record or medical claims data that partly account for the association between opioid use and new or worsening depression. Prospective studies often fail to recruit the most severely ill patients who may have more intractable pain, more severe opioid misuse, greater number of psychiatric comorbidities or more severe symptoms. If these patients do not volunteer for primary data collection, we may miss those patients with more severe conditions and at greatest risk for depression. Non-participation related to severity and clinical complexity are not a barrier to inclusion in retrospective cohort studies which could contribute to the many positive findings between LTOT and risk for depression in retrospective as compared to prospective cohort studies. In addition, retrospective studies using medical record or medical claims do not have emotional or social support measures and do not include standard measures of pain-related functional impairment. Adjusting for these factors in prospective studies may contribute to different findings between these two observational study designs.

Our findings confirm the complexity and vulnerability of patients with non-cancer pain, prescription opioid use, and depression. They suggest intensity of opioid use is not an independent predictor of depression trajectories. Instead, there appears to be numerous correlates of daily vs. non-daily opioid use such as less social support and greater pain interference that may contribute to persistent depression. Daily opioids tend to be prescribed to vulnerable patients with other risk factors for depression. The temporal order of these factors is unknown because we do not have lifetime measures of social support and pain interference. However, it is likely that there is a group in which depression preceded these factors and other cases in which worse functioning and limited emotional/social support are present prior to pain, opioid exposure, and depression. Further research is needed to disentangle these correlated factors to determine the pathways leading to depression in chronic pain and LTOT.

### 3.1. Limitations

The present results should be interpreted in the context of several limitations. First, the study was conducted at two mid-western health care systems and results may not generalize to other geographic regions. Self-reported measures may suffer from recall bias and stigma may impact reporting on prescription opioid use and depression. Patients were all starting a new period of prescription opioid use between 30 and 90 days long at baseline and patients were not required to be lifetime opioid naïve prior to this new period of opioid use. These eligibility criteria may reduce generalizability. Our follow-up period was 12-months and may not be sufficient to detect waxing and waning of depression symptoms. With longer follow-up, we may observe improvement in the stable group and worsening in the low depression group. We did not require participants to be free of depression and post-hoc analyses revealed that 24.7% of decreasing-low depression trajectory members had lifetime depression; 54.0% of the stable-moderate had lifetime depression and 59.4% of the increasing-severe depression trajectory had lifetime depression. Because prior depression episodes were more common in the more severe trajectories, it is possible that daily opioid use complicates effective depression treatment and helps sustain depression or contribute to treatment resistant depression (Scherrer, Salas, Sullivan, et al., 2016) and depression recurrence (Scherrer, Salas, Copeland, Stock, Schneider, et al., 2016). Past depression episodes are also likely a component of the complex patient characteristics that contribute to future depression. It is possible that the lack of an association between daily opioid consumption vs. non-daily opioid use and more severe or worsening depression is due to the timing of our measurements. It is possible that a prior period of prescription opioid use led to moderate depression which was present at the first, eligible monthly survey in the moderate-stable and severe-increasing trajectories. Although speculative, the opioid exposure could have led to new onset depression prior to study enrollment. In this case, it is possible that unmeasured, past opioid consumption patterns contributed to developing depression but not to the course of depression symptoms.. Lastly, FIML in GMM may underestimate worsening depression status, especially if dropout is not missing at random and related to worsening depression symptoms or pain. However, inclusion of variables in the model that may be associated with dropout (i.e. depression symptoms) improve the likelihood of meeting the MAR assumption and producing unbiased results (Jackson et al., 2019).

## 4. Conclusions

Daily prescription opioid use in this sample was not significantly associated with more severe depression trajectories. Patient characteristics concurrent or preceding the new period of prescription opioid exposure were significantly associated with depression trajectories. The lack of an independent link between daily opioid use and more severe or worsening depression in this sample suggests that increased rates of depression in daily opioid-exposed patients may be a result of the high risk patients who receive daily opioids rather than a direct effect of the daily opioid exposure. Smoking is one of the most robust risk factors for depression in patients receiving LTOT and emotional support and ability to maintain function are inversely associated with worse depression. Screening for this combination of risk factors should be part of decision making around starting and continuing opioid therapy in non-cancer pain.

## Supplementary Material

1

Supplementary material associated with this article can be found, in the online version, at doi:10.1016/j.psychres.2026.117096.

## Figures and Tables

**Table 1:** Distribution of baseline covariates (*n* = 760).

Covariates, mean(±sd) or n(%)	Overall (*n* = 760)

Age, mean (±sd)	53.6 (±11.7)
Sex	
Male	239 (31.5)
Female	521 (68.5)
Race	
Non-White	224 (29.5)
White	536 (70.5)
PROMIS - Emotional support, mean(±sd)	54.6 (±9.1)
PROMIS - Social roles, mean (±sd)	46.3 (±10.1)
# pain sites, mean (±sd)	6.1 (±3.7)
BPI1 pain severity, mean (±sd)	5.9 (±1.7)
BPI pain interference, mean (±sd)	6.7 (±2.1)
Arthritis	592 (77.9)
Back pain	466 (61.3)
Musculoskeletal pain	501 (65.9)
Fibromyalgia	58 (7.6)
Neuropathy	149 (19.6)
Chronic pain	363 (47.8)
Headache	132 (17.4)
Physical therapy	333 (43.8)
Interventional pain procedures	405 (53.3)
Antidepressants	397 (52.2)
Benzodiazepines	220 (28.9)
Gabapentin	315 (41.5)
NSAID	421 (55.4)
Muscle relaxers	443 (58.3)
Steroids	298 (39.2)
History of SUD^2^	97 (12.8)
Current smoker	180 (23.7)
PC-PTSD^3^ positive	131 (17.2)
GAD^4^ positive	163 (21.5)
PODS^5^ positive	129 (17.0)
Vital exhaustion positive	160 (21.1)
Daily opioid use	513 (67.5)

1) BPI - Brief Pain Inventory

2) SUD - substance use disorder

3) PC-PTSD - primary care PTSD screen

4) GAD - generalized anxiety disorder

5) PODS - Prescribed Opioids Difficulties Scale.

**Table 2:** PHQ-9 Score - Observed means (±sd) and estimated intercept and slope parameters for each class from GMM.

Parameter	Class 1 (*n* = 189, 24.9%) Moderate - Stable	Class 2 (*n* = 69, 9.1%) Severe - Increase	Class 3 (*n* = 502, 66.0%) Low - Decrease

Observed Month 1, mean (±sd)	12.1 (±5.6)	14.9 (±6.1)	5.2 (±4.2)
Observed Month 12, mean (±sd)	11.1 (±3.3)	20.4 (±3.8)	3.2 (±3.0)
GMM estimates	Estimate (SE, p-value)	Estimate (SE, p-value)	Estimate (SE, p-value)
Estimated Intercept	11.5 (0.7, <0.0001)	14.3 (1.2, <0.0001)	5.4 (0.3, <0.0001)
Estimated Slope	−0.03 (0.05, 0.48)	0.5 (0.1, <0.0001)	−0.2 (0.02, <0.0001)

sd=standard deviation; se=standard error; GMM=growth mixture model.

**Table 3:** Distribution of covariates by within each PHQ-9 depression trajectory (*n* = 760).

Baseline variable	Class 1 (*n* = 189) Moderate - Stable	Class 2 (*n* = 69) Severe - Increase	Class 3 (*n* = 502) Low - Decrease	p-value

Age, mean (±sd)	51.9 (±11.6)^1^	49.8 (±12.6)^1^	54.8 (±11.4)^2^	.0002
Sex				.082
Male	48 (25.4)	20 (29.0)	171 (34.1)	
Female	141 (74.6)	49 (71.0)	331 (65.9)	
Race				.141
Non-White	45 (23.8)	21 (30.4)	158 (31.5)	
White	144 (76.2)	48 (69.6)	344 (68.5)	
PROMIS - Emotional support, mean (±sd)	52.6 (±9.4)^1^	46.6 (±11.3)^2^	56.5 (±7.9)^3^	<0.0001
PROMIS - Social roles, mean (±sd)	42.0 (±8.5)^1^	40.6 (±9.7)^1^	48.7 (±9.9)^2^	<0.0001
# pain sites, mean (±sd)	7.8 (±3.8)^1^	8.9 (±3.7)^1^	5.1 (±3.1)^2^	<0.0001
BPI^1^ pain severity, mean (±sd)	6.3 (±1.6)^1^	7.0 (±1.5)^2^	5.6 (±1.7)^3^	<0.0001
BPI pain interference, mean (±sd)	7.3 (±1.8)^1^	8.2 (±1.6)^2^	6.2 (±2.1)^3^	<0.0001
Arthritis	143 (75.7)	53 (76.8)	396 (78.9)	.644
Back pain	126 (66.7)	54 (78.3)	286 (57.0)	.001
Musculoskeletal pain	124 (65.6)	45 (65.2)	332 (66.1	.983
Fibromyalgia	28 (14.8)	5 (7.3)	25 (5.0)	.0002
Neuropathy	46 (24.3)	19 (27.5)	84 (16.7)	.018
Chronic pain	93 (49.2)	39 (56.5)	231 (46.0)	.235
Headache	42 (22.2)	18 (26.1)	72 (14.3	.007
Physical therapy	70 (37.0)	29 (42.0)	234 (46.6)	.074
Interventional pain procedures	101 (53.4)	41 (59.4)	263 (52.4)	.547
Antidepressants	133 (70.4)	37 (53.6)	227 (45.2)	<0.0001
Benzodiazepines	67 (35.5)	26 (37.7)	127 (25.3)	.008
Gabapentin	95 (50.3)	33 (47.8)	187 (37.3)	.004
NSAID	95 (50.3)	41 (59.4)	285 (56.7)	.240
Muscle relaxers	114 (60.3)	44 (63.8)	285 (56.8)	.439
Steroids	86 (45.5)	26 (37.7)	186 (37.1)	.123
History of SUD^2^	28 (14.8)	12 (17.4)	57 (11.4)	.230
Current smoker	61 (32.3)	28 (40.6)	91 (18.1)	<0.0001
PC-PTSD^3^ positive	62 (32.8)	27 (39.1)	42 (8.4)	<0.0001
GAD^4^ positive	71 (37.6)	39 (56.5)	53 (10.6)	<0.0001
PODS^5^ positive	46 (24.3)	15 (21.7)	68 (13.6)	.002
Vital exhaustion positive	72 (38.1)	42 (60.9)	46 (9.2)	<0.0001
Daily opioid use	133 (70.4)	48 (69.6)	332 (66.1)	.530

Means with the same subscript are not significantly different - Tukey’s post-hoc.

1) BPI - Brief Pain Inventory

2) SUD - substance use disorder

3) PC-PTSD - primary care PTSD screen

4) GAD - generalized anxiety disorder

5) PODS - Prescribed Opioids Difficulties Scale.

**Table 4:** Distribution of baseline covariates, by daily opioid use (*n* = 760).

Covariates	No Daily Opioid (*n* = 247)	Daily Opioid (*n* = 513)	p-value

Age, mean (±sd)	54.1 (±12.0)	53.4 (±11.5)	.406
Sex			.009
Male	62 (25.1)	177 (34.5)	
Female	185 (74.9)	336 (65.5)	
Race			.016
Non-White	87 (35.2)	137 (26.7)	
White	160 (64.8)	376 (73.3)	
PROMIS - Emotional support, mean(±sd)	55.5 (±8.7)	54.2 (±9.3)	.073
PROMIS - Social roles, mean (±sd)	48.2 (±10.4)	45.4 (±9.9)	.0002
# pain sites, mean (±sd)	5.6 (±3.5)	6.3 (±3.7)	.022
BPI^1^ pain severity, mean (±sd)	5.6 (±1.7)	6.0 (±1.7)	.0007
BPI pain interference, mean (±sd)	6.1 (±2.2)	6.9 (±2.0)	<0.0001
Arthritis	200 (81.0)	392 (76.4)	.156
Back pain	133 (53.8)	333 (64.9)	.003
Musculoskeletal pain	144 (58.3)	357 (69.6)	.002
Fibromyalgia	20 (8.1)	38 (7.4)	.737
Neuropathy	41 (16.6)	108 (21.1)	.147
Chronic pain	113 (45.7)	250 (48.7)	.440
Headache	39 (15.8)	93 (18.1)	.425
Physical therapy	62 (25.1)	271 (52.8)	<0.0001
Interventional pain procedures	104 (42.1)	301 (58.7)	<0.0001
Antidepressants	128 (51.8)	269 (52.4)	.874
Benzodiazepines	62 (25.1)	158 (30.8)	.105
Gabapentin	69 (27.9)	246 (48.0)	<0.0001
NSAID	126 (51.0)	295 (57.5)	.092
Muscle relaxers	110 (44.5)	333 (64.9)	<0.0001
Steroids	102 (41.3)	196 (38.2)	.414
History of SUD^2^	14 (5.7)	83 (16.2)	<0.0001
Current smoker	37 (15.0)	143 (27.9)	<0.0001
PC-PTSD^3^ positive	38 (15.4)	93 (18.1)	.348
GAD^4^ positive	48 (19.4)	115 (22.4)	.348
PODS^5^ positive	20 (8.1)	109 (21.3)	<0.0001
Vital exhaustion positive	50 (20.2)	110 (21.4)	.704

1) BPI - Brief Pain Inventory

2) SUD - substance use disorder

3) PC-PTSD - primary care PTSD screen

4) GAD - generalized anxiety disorder

5) PODS - Prescribed Opioids Difficulties Scale.

**Table 5:** Multivariable, multinomial logistic regression, Class 3 (Low-Decrease) as common outcome referent. Covariates included are those with both *p* < 0.10 for: a) for PHQ-9 bivariate association and b) bivariate association with daily opioid (*n* = 760).

Baseline variable	Class 1 Moderate-Stable vs. Class 3 Low-Decrease OR (95% CI)	Class 2 Severe-Increase vs. Class 3 Low-Decrease OR (95% CI)

Daily opioid	0.87 (0.56–1.36)	0.56 (0.29–1.09)
Sex		
Male	1.00	1.00
Female	1.42 (0.93–2.18)	1.22 (0.64–2.34)
PROMIS - Emotional support	0.96 (0.94–0.99)	0.92 (0.89–0.94)
PROMIS - Social roles	0.95 (0.93–0.97)	0.96 (0.93–0.99)
# pain sites	1.17 (1.10–1.24)	1.20 (1.10–1.31)
BPI^1^ pain severity	1.03 (0.89–1.19)	1.19 (0.95–1.49)
BPI pain interference	1.07 (0.94–1.21)	1.39 (1.11 –1.74)
Back pain	0.98 (0.65–1.48)	1.78 (0.89–3.53)
Physical therapy	0.55 (0.36–0.84)	0.90 (0.47–1.73)
Gabapentin	1.61 (1.07–2.42)	1.11 (0.60–2.05)
Current smoker	1.76 (1.14–2.72)	2.38 (1.27–4.47)
PODS2 positive	1.66 (1.02–2.71)	1.17 (0.56–2.46)

OR=odds ratio; CI=confidence interval.

1) BPI - Brief Pain Inventory

2) PODS - Prescribed Opioids Difficulties Scale.
