# Supplementary material for: Prescription opioid use and 12-month depression trajectories
Source: Psychiatry Res. Author manuscript; Available in PMC 2026 Jun 15. (PMC13267013; doi:10.1016/j.psychres.2026.117096)
Supplement: 1 [file NIHMS2180152-supplement-1.docx]

| **e-table 1. ICD-10-CM and CPT codes for EHR related variables** | |
| --- | --- |
| Arthritis | ICD-10 code: M00.x to M02.x, M05.x, M06.x, M08.x, M12.x to M19.x, M23.x, M24.0x to M24.4x, M24.6x to M24.9, M25.x, M32.10, M33.20, M33.90, M34.0, M34.1, M34.9, M35.00, M35.01, M35.5, M35.9, M36.2, M36.3, M36.4, M43.4, M43.5x, M45.9, M79.6x, R26.2, R29.4, R29.898, Z87.39 |
| Musculoskeletal pain | ICD-10 code: M35.3, M60.x to M79.x (**excluding M79.7**), R29.898, R29.91, S03.x, S13.x, S16.x, S23.x, S33.x, S39.0x, S39.9x, S43.x, S46.x, S53.x, S56.x, S63.x, S66.x, S73.x, S76.x, S83.x, S86.x, S93.x, S96.x, Z96.6x, Z97.1x, Z89.x |
| Back and Neck pain | ICD-10 code: M43.2x, M43.6, M43.8x, M43.9, M46.0x, M46.1, M46.4x, M46.8x, M46.9x, M47.x, M48.0x, M48.1x, M48.2x, M48.3x, M48.8x, M48.9, M49.8x, M50.x to M51.x, M53.x to M54.x, M96.1, Q76.0 to Q76.3, Q76.4x, Q76.6 |
| fibromyalgia | ICD10 – M79.7 |
| Chronic pain | ICD-10: G89.2x |
| Neuropathy | ICD-10 code: B02.23, B26.84, G90.0x, G99.0, G54.x to G65.x |
| Headache | ICD-10 code: G43.x, G44.x, R51.x |
| Physical Therapy | CPT code: 97010 – 97039; 97110 – 97150; 97530 – 97546; 97161 – 97164; 97750 – 97755 |
| Interventional pain procedures | CPT code: 20526, 20527, 23350, 27093, 27096, 77002, 77003, 96372, 95874, J7321, J7323, J7324, J7325, J7326, J0585, J0587, 64505, 64510, 64517, 64520, 64530;  20550-20553; 20600-20611; 62280-62287; 62310-62327; 62350-62365; 63650-63655; 63663-63688; 64479-64495; 64633-64636; 64460-64659; 64400-64455; 22510-22515; 64612-64615 |

**APPENDIX**

| Appendix e-Table 2. Distribution of baseline covariates – One-sample z- or t-tests comparing distribution of variables among those with 3 or more monthly surveys to those in the entire sample | | | |
| --- | --- | --- | --- |
| **Covariates, mean(±sd) or %** | **Entire sample**  **(n=1047)** | **3+ monthly**  **(n=842)** | **One-sample p-value*** |
| Age, mean (±sd) | 52.8 (±11.7) | 53.4 (±11.8) | .111 |
| Sex: Female | 67.24 | 69.0 | .275 |
| Race: White | 70.42 | 70.6 | .926 |
| PROMIS – Emotional support, mean(±sd) | 54.6 (±11.9) | 54.5 (±9.3) | .703 |
| PROMIS – Social roles, mean (±sd) | 46.4 (±9.9) | 46.3 (±10.0) | .730 |
| # pain sites, mean (±sd) | 6.2 (±3.7) | 6.1 (±3.7) | .589 |
| BPI^1^ pain severity, mean (±sd) | 5.9 (±1.8) | 5.9 (±1.7) | .989 |
| BPI pain interference, mean (±sd) | 6.7 (±2.2) | 6.7 (±2.2) | .825 |
| Daily opioid use | 67.7 | 67.9 | .932 |
| Arthritis | 75.9 | 77.1 | .436 |
| Back pain | 58.9 | 60.6 | .333 |
| Musculoskeletal pain | 65.9 | 65.9 | .993 |
| Fibromyalgia | 8.1 | 7.8 | .765 |
| Neuropathy | 19.4 | 20.2 | .557 |
| Chronic pain | 45.6 | 46.7 | .516 |
| Headache | 17.1 | 18.0 | .463 |
| Physical therapy | 45.5 | 43.9 | .377 |
| Interventional pain procedures | 50.6 | 52.0 | .417 |
| Antidepressants | 50.5 | 51.9 | .426 |
| Benzodiazepines | 28.8 | 28.7 | .949 |
| Gabapentin | 41.9 | 42.0 | .947 |
| NSAID | 52.0 | 54.3 | .179 |
| Muscle relaxers | 58.5 | 58.0 | .727 |
| Steroids | 38.0 | 38.5 | .779 |
| History of SUD^2^ | 12.2 | 12.0 | .893 |
| Current smoker | 26.8 | 23.4 | .028 |
| PC-PTSD^3^ positive | 18.1 | 17.9 | .892 |
| GAD^4^ positive | 22.1 | 21.7 | .769 |
| PODS^5^ positive | 16.8 | 17.2 | .753 |
| Vital exhaustion positive | 20.5 | 21.3 | .603 |
| \| 1) BPI – Brief Pain Inventory; 2) SUD – substance use disorder; 3)PC-PTSD – primary care PTSD screen; 4) GAD – generalized anxiety disorder; 5) PODS – Prescribed Opioids Difficulties Scale \| \| --- \| | | | |

| **Appendix e-table 3.** Distribution of type of opioid at baseline (not mutually exclusive) (n=760) | |
| --- | --- |
| **Type of opioid** | **n (%)** |
| Codeine | 75 (9.9) |
| Tramadol | 291 (38.3) |
| Hydrocodone | 370 (48.7) |
| Oxycodone | 234 (30.8) |
| Morphine | 19 (2.5) |
| Methadone | < 5 |
| Fentanyl | < 5 |
| Dihydrocodeine | 0 (0.0) |
| Hydromorphone | 8 (1.1) |
| Levorphanol | 0 (0.0) |
| Meperidine | < 5 |
| Oxymorphone | 0 (0.0) |
| Pentazocine | < 5 |
| Tapentadol | < 5 |
|  |  |
| > 2 opioid types | 201 (26.4) |
| *cells with < 5 participants are suppressed to protect confidentiality | |

| **Appendix e-Table 4. Model fit statistics for Growth Mixture Modeling results of monthly PHQ-9 Scores (n= 760)** | | | | | | |  |
| --- | --- | --- | --- | --- | --- | --- | --- |
| Classes | AIC | BIC | aBIC | Entropy | LMR | Final class proportions | |
| 1 | 35016.6 | 35095.3 | 35041.4 | --- | --- | 1.00 | |
| 2 | 34875.5 | 34968.2 | 34904.6 | 0.81 | 0.0003 | 82.5%; 17.5% | |
| **3** | **34831.5** | **34938.1** | **34865.1** | **0.76** | **0.007** | **24.9%; 9.1%; 66.0%** | |
| 4 | 34789.7 | 34910.2 | 34827.7 | 0.80 | 0.10 | 73.1%; 14.2%; 8.6%; 4.1% | |
| Note: Bolded model selected as best fitting model.  #par = number of parameters estimated; LL=log likelihood; AIC=Akaike information criterion; BIC=Bayesian information criterion; aBIC=adjusted Bayesian information criterion; LMR = Lo-Mendell-Rubin adjusted likelihood ratio test comparing solution for k vs. k-1 classes | | | | | | |  |
